# Headliners: Cancer: Inhibition of RLIP76 Causes Complete Regression of Melanoma in Mice

**Published:** 2006-05

**Authors:** Jerry Phelps

Singhal SS, Awasthi YC, Awasthi S. 2006. Regression of melanoma in a murine model by RLIP76 depletion. Cancer Res 66:2354–2360.

Studies have shown that inhibition or depletion of RLIP76, a glutathione-conjugate transport protein that helps cells defend themselves against toxicants, causes apoptosis in a number of cancer cell types. Now NIEHS-supported researcher Yogesh C. Awasthi of The University of Texas Medical Branch at Galveston and colleagues have confirmed that inhibition or depletion of RLIP76 causes apoptosis in malignant melanoma cells.

RLIP76 is implicated in the regulation of multiple signaling pathways. The clinical and physiological implications of RLIP76 extend to diverse processes, including stress resistance, chemotherapy drug resistance, radiation resistance, oxidative stress–induced disease, and even insulin resistance.

The Texas researchers compared the expression of RLIP76 in normal cells and several cancer cell lines to explore potential clinical impacts. Their studies also included techniques to determine whether depletion of RLIP76 would cause cancer-specific apoptosis. Expression of RLIP76 was found to be greater in malignant cells than in nonmalignant cells. Inhibition or depletion of the protein also caused preferential apoptosis in a variety of malignant cells in culture. Most importantly, in a mouse melanoma model, administration of a single dose of RLIP76 antibodies, short interfering RNAs, or antisense oligonucleotides caused complete tumor regression in 10 days.

These findings provide strong evidence that inhibition of RLIP76 through genetic engineering or by administration of antibodies may be a clinically relevant approach to treating cancer, especially melanoma. The dramatic results suggest advancing this technique to clinical practice. Further studies in melanoma and other cancer models and other susceptible cancer cell lines would be needed to show the general applicability of these results prior to human clinical applications.

## Figures and Tables

**Figure:**
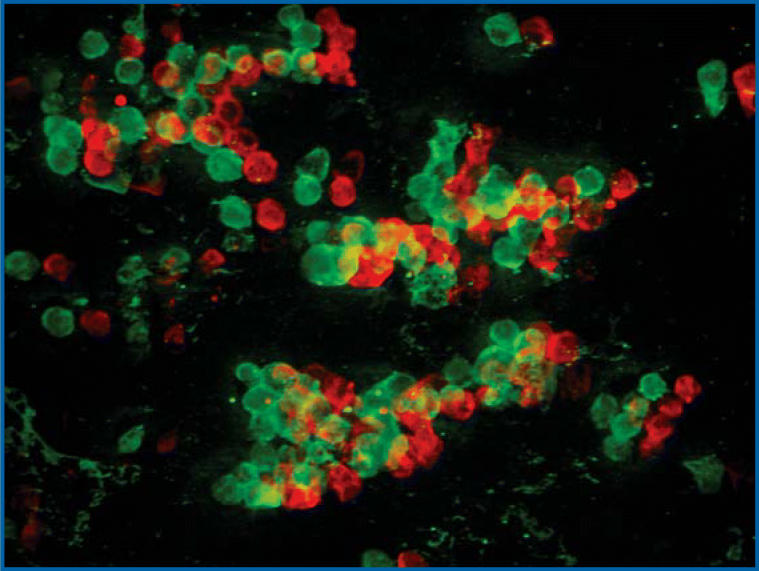
Apoptotic (green) melanoma cells after treatment

